# Response to “Categorization of post-cardiac arrest patients according to the pattern of amplitude-integrated electroencephalography after return of spontaneous circulation”

**DOI:** 10.1186/s13054-018-2252-1

**Published:** 2018-12-18

**Authors:** Jia Li, Ning-Tao Li, Yong-Gang Peng

**Affiliations:** 1 Department of Anesthesiology, Affiliated Cancer Hospital of Zhengzhou University, Henan Cancer Hospital, Zhengzhou, 450003 China; 2 Department of Anesthesiology, Henan Provincial People’s Hospital, Zhengzhou, 450000 China; 3 Department of Anesthesiology, University of Florida, Gainesville, FL 32610-0254 USA; 4 Department of Anesthesiology, Henan Provincial People’s Hospital, NO 7, WeiWu Road, Zheng zhou, 210009 China

We read with great interest the recent article by Sugiyama et al. [1], “Categorization of post-cardiac arrest patients according to the pattern of amplitude-integrated electroencephalography after return of spontaneous circulation.” However, we have some concerns in regards to their study conclusion. The authors claimed that they can successfully categorize patient’s neurological prognoses according to their pattern of electroencephalography (EEG) during the early post-cardiac arrest period, which could be used as a potential guide to customize post-cardiac arrest care for each patient.

Early studies have shown that reductions in core temperature during therapeutic hypothermia (TH) cause vaso-constriction and shivering, which result in skin breakdown, infection, and increased oxygen consumption [2]. To prevent the above observed complications, anesthetics and sedatives were administered during TH in adult and pediatric patients after cardiac arrest (CA).

EEG has exhibited a direct reflection of the effects of sedatives and anesthetics on patients and offers unique guidance on the adequacy of anesthesia [3]. Under such circumstances, an important question is raised: the EEG may not reflect the patient’s true neurological status during the phase of mild TH with sedative and anesthetic intervention after CA.

The EEG pattern could be inadvertently altered by sedatives and anesthetics, and not be the native representation of patient brain function during the interventional period of mild TH. In addition, it is still debatable how to precisely titrate the depth of sedation during TH for patients after CA. Furthermore, the pharmacokinetics of sedative regimens may be altered under mild hypothermia and result in prolonged systemic clearance of anesthetic agents. There may be accumulative effects of sedatives for CA patients who have undergone TH, which leads to delay in awakening, extending the duration of mechanical ventilation and other subsequent complications [4]. Mild TH plus the cumulative effect of sedatives could have a significant impact on the activity of brain function; one would suspect that the predictive value of the EEG could be obscured [5]. Therefore, when sedatives and anesthetics are given to patients during TH after CA, the EEG is not consistent and its predictive value becomes questionable.

From the discussion above, the current study by Sugiyama et al. did not provide convincing evidence that the EEG pattern has reliable value in predicting a patient’s neurological prognosis when influenced by hypothermia and sedative agents. We suggest that further study is necessary to elucidate the relationship between EEG and neurological outcome after CA.
