# Supplementary material for: Response to “Categorization of post-cardiac arrest patients according to the pattern of amplitude-integrated electroencephalography after return of spontaneous circulation”
Source: Crit Care. 2018 Dec 18;22:346. doi: 10.1186/s13054-018-2252-1 (PMC6299588; doi:10.1186/s13054-018-2252-1)
Supplement: Related texts

# Reply: Authors’ response

**Authors:** Kazuhiro Sugiyama

We would like to thank Dr. Li and colleagues for their letter.

They comment on the influence of sedatives during target temperature management (TTM) on EEG. However, the administration of sedatives during TTM is currently required for any prognostic study after cardiac arrest, and many studies have reported the usefulness of continuous EEG in this field [6, 7].

Our study showed that post-cardiac arrest patients could be categorized according to their neurological prognosis using the recovery time to continuous normal voltage and the presence of burst suppression in amplitude-integrated EEG (aEEG) monitoring. In our study, 0.05–0.1 mg/kg/h midazolam was used for sedation and a muscle relaxant was used to control shivering. In another study by Oh et al. evaluating the pattern of aEEG in post cardiac patients, 0.04–0.2 mg/kg/h midazolam and a muscle relaxant were also administered [8]. The cut-off for continuous normal voltage (CNV) recovery time for predicting good neurological outcome was around 24 h in both studies [8, 9]. Furthermore, although the prolonged sedation caused by accumulation of medication could affect the CNV recovery time, a recovery time of more than 36 h predicted poor neurological outcomes in both studies. In the sedation protocol with the administration of this dose of midazolam and a muscle relaxant, the categorization suggested in our study could contribute to precise prognostication and determination of the severity of hypoxic encephalopathy.

However, as Dr. Li pointed out, the sedation protocol during TTM remained undetermined. If propofol or a higher dose of midazolam is used for sedation during TTM, the CNV recovery time might be affected. To our knowledge, no prior study evaluated the difference in CNV recovery time among different sedatives in post-cardiac arrest patients. Furthermore, the meaning of burst suppression might be different between midazolam and propofol. As we discussed in the article, burst suppression can be induced by propofol, and might lose its meaning as a predictor of neurological prognosis in a sedation protocol using propofol.

We believe it is worth investigating whether the categorization in our study can predict prognosis in post-cardiac patients with good accuracy using different sedation protocols during TTM.
